# Components of the Plasminogen Activation System Promote Engraftment of Porous Polyethylene Biomaterial via Common and Distinct Effects

**DOI:** 10.1371/journal.pone.0116883

**Published:** 2015-02-06

**Authors:** Christoph A. Reichel, Maximilian E. T. Hessenauer, Kerstin Pflieger, Markus Rehberg, Sandip M. Kanse, Stefan Zahler, Fritz Krombach, Alexander Berghaus, Sebastian Strieth

**Affiliations:** 1 Department of Otorhinolaryngology, Head and Neck Surgery, Ludwig-Maximilians-Universität München, Munich, Germany; 2 Walter Brendel Centre of Experimental Medicine, Ludwig-Maximilians-Universität München, Munich, Germany; 3 Department of Pharmacy, Ludwig-Maximilians-Universität München, Munich, Germany; 4 Institute of Basic Medical Sciences, University of Oslo, Oslo, Norway; 5 Department of Otorhinolaryngology, Head and Neck Surgery, Johannes Gutenberg University Medical Center, Mainz, Germany; University of Pécs Medical School, HUNGARY

## Abstract

Rapid fibrovascularization is a prerequisite for successful biomaterial engraftment. In addition to their well-known roles in fibrinolysis, urokinase-type plasminogen activator (uPA) and tissue plasminogen activator (tPA) or their inhibitor plasminogen activator inhibitor-1 (PAI-1) have recently been implicated as individual mediators in non-fibrinolytic processes, including cell adhesion, migration, and proliferation. Since these events are critical for fibrovascularization of biomaterial, we hypothesized that the components of the plasminogen activation system contribute to biomaterial engraftment. Employing *in vivo* and *ex vivo* microscopy techniques, vessel and collagen network formation within porous polyethylene (PPE) implants engrafted into dorsal skinfold chambers were found to be significantly impaired in uPA-, tPA-, or PAI-1-deficient mice. Consequently, the force required for mechanical disintegration of the implants out of the host tissue was significantly lower in the mutant mice than in wild-type controls. Conversely, surface coating with recombinant uPA, tPA, non-catalytic uPA, or PAI-1, but not with non-catalytic tPA, accelerated implant vascularization in wild-type mice. Thus, uPA, tPA, and PAI-1 contribute to the fibrovascularization of PPE implants through common and distinct effects. As clinical perspective, surface coating with recombinant uPA, tPA, or PAI-1 might provide a novel strategy for accelerating the vascularization of this biomaterial.

## Introduction

In reconstructive medicine, biomaterials are used for the augmentation or replacement of tissues [1]. If autologous transplant materials are not sufficiently available, reconstructive options for bone and cartilage tissue defects include the use of porous polyethylene biomaterial (PPE) implants [1]. PPE implants are available for numerous applications ranging from orbital floor reconstruction [2] over auricular reconstruction [3] to facial contour implants [4]. This implant material has proofed to be biocompatible, long-term stable, non-soluble, and non-resorbable over a period of more than 30 years of clinical use [5–7]. However, there are applications which are less favorable for implant integration, such as rhinoplasty and reconstructive nose surgery [8] as well as reconstructions in irradiated or scar tissue, where blood supply is weak [9].

A ‘moderate’ inflammatory response upon implantation, which induces only transient and limited changes in microvascular permeability and alterations in leukocyte-endothelial cell interactions, as well as the rapid formation of a dense vessel and collagen network within the implant are considered to be prerequisites for successful biomaterial engraftment [10,11]. Interference with or delay of these events promote implant infection and dislocation, which ultimately might result in implant rejection [10,11].

Fibrinolysis is a fundamental biological process, which maintains tissue perfusion by preventing clot formation in blood vessels [12,13]. Plasmin is the key enzyme in the fibrinolytic system, which mediates the dissolution of fibrin polymers [14,15]. Its zymogen plasminogen is activated by tissue plasminogen activator (tPA) [16] and–to a lesser degree–by urokinase-type plasminogen activator (uPA) [13,17]. The activity of these serine proteases is tightly controlled by plasminogen activator inhibitor-1 (PAI-1) [18]. These components of the plasminogen activation system are expressed by a variety of cell types, including endothelial cells [19–21]. In addition to their well-known roles in fibrinolysis, uPA, tPA, and PAI-1 are increasingly recognized as individual mediators in non-fibrinolytic processes, such as cell adhesion, migration, and proliferation [22–30]. Since these events are critical for the fibrovascularization of biomaterial, we hypothesized that the plasminogen activation system is involved in the biointegration of PPE implants.

In the present study, we demonstrate that endogenously released uPA, tPA, and PAI-1 contribute to the functional biointegration of PPE implants via common and distinct effects: Whereas tPA predominantly facilitates the initial vessel sprouting into PPE biomaterial, uPA additionally exhibits sustained effects on vascularization of these implants. Interestingly, PAI-1, which serves as the principal inhibitor of tPA and uPA in fibrinolysis, supports vascularization of PPE biomaterial differentially at various time points after implantation. Moreover, the major components of the plasminogen activation system were found to be critically involved in collagen deposition within PPE biomaterial thereby promoting mechanical tissue integration of the implants. Notably, uPA, tPA, and PAI-1 exhibited strong chemotactic effects on endothelial cells. Accordingly, surface coating with recombinant uPA (mainly via its non-proteolytic properties) and–to a lesser degree–with tPA (mainly via its proteolytic properties) or PAI-1 accelerated vascularization of PPE implants. Our experimental findings provide novel insights into the mechanisms underlying tissue integration of biomaterials and propose surface coating with recombinant uPA, tPA, or PAI-1 as a novel promising strategy for accelerating the vascularization of PPE implants under unfavorable conditions.

## Materials and Methods

### Animals

All animal experiments were performed according to German legislation for the protection of animals and approved by the Regierung von Oberbayern, München, Germany. Protocols were approved under permit 55.2-1-54-2531-115-10.

Female C57BL/6J mice were purchased from Charles River (Sulzfeld, Germany). Female uPA^-/-^, tPA^-/-^, and PAI-1^-/-^ mice were generated as described previously and backcrossed on the C57BL/6J background for 10 generations [31,32]. All experiments were performed with mice at the age of 8–16 weeks. Animals were housed in single cages and had free access to tap water and standard laboratory food (ssniff, Spezialdiaeten GmbH, Soest, Germany) throughout the experiments.

### Dorsal skinfold chamber model

The surgical preparation of the dorsal skinfold chamber was performed as described previously [11,33]. Briefly, two symmetrical titanium frames were implanted on the extended dorsal skinfold thereby sandwiching the double layer of the shaved and depilated skin. Subsequently, one layer of skin–consisting of epithelial skin layer, subcutaneous tissue, and striated skin muscle–was completely removed in a circular area of about 15 mm in diameter and the remaining contralateral layers are covered with a removable coverslip incorporated into the observation window of the titanium frame. All surgical procedures were performed under anesthesia with ketamine (75 mg/kg b.w. i.p., Ketavet; Parke-Davis, Berlin, Germany) and xylazine (25 mg/kg b.w. i.p., Rompun; Bayer, Leverkusen, Germany). After surgery, animals were allowed to recover for a period of 48 hours from anesthesia and microsurgery. Only chamber preparations fulfilling the criteria of microscopically intact microcirculation and no signs of inflammation were included in the experiments. For the implantation of the PPE material, the coverslip of the skinfold chamber was temporarily removed and the PPE material was implanted into the striated skin muscle. The chambers were well tolerated and the animals showed no signs of discomfort, as indicated by normal feeding and sleeping habits. At the end of the experiment, animals were euthanized by an intraperitoneal injection of pentobarbital (Narcoren, Merial, Germany).

### Preparation of PPE implants

Porous polyethylene sheets (PPE; Medpor; PorexSurgical, Newnan, GA; pore size 100–250 μm) were cut into 3.0 x 3.0 x 0.1 mm slices and subsequently steam sterilized. Before implantation, the PPE material was either bathed in sterile 0.9% saline solution or coated as described previously [34] using 50 μL growth factor reduced (GFR) BD Matrigel Matrix (Becton Dickinson, Heidelberg, Germany) or GFR BD Matrigel Matrix supplemented with 1 μg of recombinant murine uPA, diisopropyl fluorophosphate (DFP)-uPA, tPA, non-enzymatic (NE)-tPA, or PAI-1 (Molecular Innovations, Novi, MI). Liquid GFR BD Matrigel entered the PPE material thoroughly at 4°C and was allowed to polymerize for 1h at 37°C before implantation.

### In vivo fluorescence microscopy

For *in vivo* fluorescence microscopy, the awake chamber-bearing mice were immobilized in a Perspex tube on a custom-made stage (Effenberger, Munich, Germany) under a modified Zeiss microscope (Axiotech Vario; Zeiss, Göttingen, Germany). Fluorescein isothiocyanate (FITC)-labeled dextran (Sigma, Deisenhofen, Germany; MW 500,000; 0.05–0.1 mL of a 5% solution in 0.9% saline) was used to enhance the contrast of the vascular network and rhodamine 6G (Molecular Probes, Eugene, OR; 0.04 mL of a 0.05% solution in 0.9% saline) was used to visualize leukocyte-endothelial cell interactions. The fluorescent dyes were administered via tail vein injection. Selective observation of FITC-labeled plasma and rhodamine 6G-labeled leukocytes was possible using epi-illumination with a 100 W mercury lamp with selective filter blocks (Zeiss, Göttingen, Germany).

### Microcirculatory analysis

In each experiment, 9 regions of interest (ROI) were selected following a given scheme: 1 ROI in the center of the implant, 1 ROI in each corner of the rectangular implant and 4 ROIs in the surrounding host tissue. These ROIs were examined on day 7, 10, and 14 after biomaterial implantation. *In vivo* fluorescence microscopy images were acquired by a charge-coupled device (CCD) camera (Sony XC-77CE; Sony, Cologne, Germany) and recorded on digital tapes (Sony DVCAM DSV 45P; Sony, Cologne, Germany) for subsequent off-line analysis (Cap Image; Zeintl, Heidelberg, Germany). Parameters for offline analysis included absolute and functional vessel density, vessel diameters, and blood flow velocities. Furthermore, skeletonized maps of the vessel network were analyzed using the ImageJ plugin ‘Analyze Skeleton’, quantifying numbers of vessel junctions, numbers of vessel branches, and average branch lengths. Leukocyte-endothelial cell interactions were characterized as numbers of rolling (< 50% of red blood cell velocity) cells crossing a given line in 30 sec and firmly adherent (for > 30 sec) cells on 100 μm of vessel wall.

### Dynamic breaking strength

Mechanical integration of the implant material into the host tissue was assessed on day 14 after implantation by measuring the force required for mechanical removal of the implant out of the host tissue using a newton meter as described previously [34].

### Collagen deposition

Collagen deposition within the pores of the implant was analyzed using multi-photon microscopy of fixed tissue samples (TriMScope, LaVision BioTec, Bielefeld, Germany) as described elsewhere [35]. Images were aquired using an XLUMPlanFl 20 x/0.95 W objective. Excitation wavelength was 1275 nm, with a laser intensity of 20 mW. The second harmonic (SHG) signal was detected using a 624/40 filter. Optical sections (500 μm x 500 μm, 1450 px) were scanned at 4 μm distances using the same settings for all samples analyzed. Z- stacks covering 100 μm were projected and further processed using ImageJ software. The second harmonic generation (SHG) signal was measured in 3 ROIs within the pores of each implant and normalized to the background signal.

### Confocal microscopy

Implants were fixed with 4% paraformaldehyde, permeabilized with 0.02% Triton X-100 (Merck, Darmstadt, Germany) and blocked with 1% BSA for 24 hours at 4°C. Subsequently implants were incubated with primary antibodies against uPA, tPA, PAI-1 (Santa Cruz, Dallas, TX), and CD31 for 72 hours at 4°C. Secondary antibodies (Alexa Fluor 488 goat anti-rat IgG, Alexa Fluor 680 goat anti-rat IgG; Invitrogen, Carlsbad, CA) were incubated for 72 hours at 4°C. Images were obtained using a SP8 SMD confocal microscope (Leica, Wetzlar, Germany) and a 63 x HC PL APO 1.2 NA water objective.

### Cell migration

Chemotaxis experiments were performed in a chemotaxis slide (Ibidi, Martinsried, Germany). Microscopy was performed at 37°C and 5% CO_2_ on an inverted microscope Eclipse Ti (Nikon, Duesseldorf, Germany), a 4 x objective and a CCD camera. Images were obtained over a total time of 20 hours every ten minutes.

HUVECs (Human umbilical vein endothelial cells from Promocell, Heidelberg, Germany) were cultured in growth medium (ECGM, Promocell, Heidelberg, Germany) containing 10% fetal calf serum. After reaching confluency, cells were trypsinised, embedded into 30% Matrigel (Corning, Amsterdam, Netherlands), diluted with basal medium (ECBM, Promocell, Heidelberg, Germany) and finally filled into the chemotaxis chambers at a density of 3 x 10^6^ cells/mL. ECGM was used as attractant-free medium, while ECGM supplemented with 30 ng/mL recombinant human tPA, 20 ng/mL uPA, or 1 μg/mL PAI-1 (Sino Biological, Beijing, China) was used to analyze the chemotactic effect of these proteins on migration of HUVECs.

Cell tracking was performed using ImageJ software (National Institutes of Health, Bethesda, USA) plugin “Manual Tracking”. 30 cells per experiment were tracked and each experiment was repeated independently three times. For further analysis and evaluation of chemotactical processes “Chemotaxis and Migration Tool” (ibidi, Martinsried, Germany) was used.

### Experimental protocol

In a first set of experiments, PPE implants were engrafted into dorsal skinfold chambers in wild-type (WT), uPA^-/-^, tPA^-/-^, and PAI-1^-/-^ mice (n = 6 per group). In a second set of experiments, implants coated with Matrigel as well as with Matrigel supplemented with murine uPA, DFP-uPA, tPA, NE-tPA, or PAI-1 were implanted into dorsal skinfold chambers in WT mice (n = 5 or 6 per group).

*In vivo* fluorescence microscopy was performed on days 7, 10, and 14 after implantation. Mice were sacrificed via terminal injection of 200 mg/kg pentobarbital, and immediately afterwards the measurement of the dynamic breaking strength was performed.

### Statistical analysis

Data analysis was performed with a statistical software package (SigmaStat for Windows, Jandel Scientific, Erkrath, Germany). The ANOVA on ranks test followed by the Dunnett test was used for the estimation of stochastic probability in intergroup comparisons. Mean values and SEM are given. P values < 0.05 were considered significant.

## Results

### Expression of uPA, tPA, and PAI-1 in PPE implants

**Figure 1:**
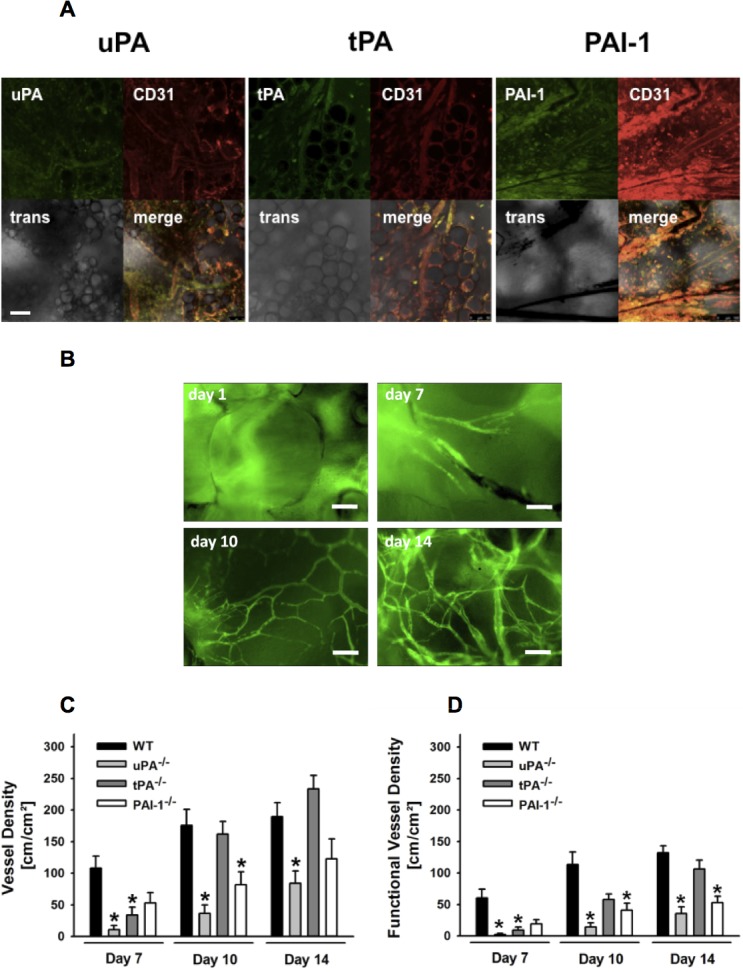
Role of endogenous uPA, tPA, and PAI-1 for vascularization of PPE biomaterial. The distribution of uPA, tPA, and PAI-1 in engrafted PPE biomaterial was analyzed *ex vivo* in implants from WT mice by confocal microscopy (**A;** scale bar: 100 μm). Vascularization of PPE implants was analyzed in WT as well as in uPA-, tPA-, or PAI-1-deficient mice by *in vivo* fluorescence microscopy. Representative *in vivo* microscopy images of PPE biomaterial vascularization in a WT mouse are shown (**B**; scale bar: 100 μm). Panels show results for the absolute (**C**) and functional (**D**) vessel density within the implant (mean ± SEM for n = 6; * p < 0.05 vs. WT).

Since plasmin(ogen) has previously been implicated in tissue integration of biomaterial [36], we hypothesized that components of the plasminogen activation system are also involved in the biointegration of these implants. Using immunostaining and confocal microscopy, expression of uPA, tPA, and PAI-1 was detected in CD31^+^ microvascular endothelial cells as well as in and around the newly formed microvessels within the implant (Fig. 1A).

### Role of uPA, tPA, and PAI-1 for vascularization of PPE implants

Rapid vascularization of PPE biomaterial is a prerequisite for its successful tissue integration [10,11]. To evaluate the functional relevance of uPA, tPA, and PAI-1 for vascularization of PPE implants, vascularization of this biomaterial was analyzed on days 7, 10, and 14 after implantation into dorsal skinfold chambers in WT, uPA^-/-^, tPA^-/-^, or PAI-1^-/-^ mice by *in vivo* fluorescence microscopy. In WT animals, initial vessel formations within the PPE implants, which were only partially perfused, were found on day 7 after implantation. On day 10 after implantation, a further densification of the vessel network was observed, whereas on day 14 after implantation, a complete network of perfused vessels was established in the PPE implants (Fig. 1B). Whereas in tPA-deficient animals absolute (Fig. 1C; representing the entire vessel network including perfused and non-perfused vessels) and functional (Fig. 1D; representing only the network of perfused vessels) vessel density were significantly reduced in the initial phase of biomaterial engraftment, PAI-1-deficient animals exhibited a significant defect in absolute vessel density at day 10 and in functional vessel density at days 10 and 14 after implantation. In uPA-deficient animals, however, absolute and functional vessel density of PPE implants were significantly diminished during the entire experimental period as compared to WT controls.

**Figure 2:**
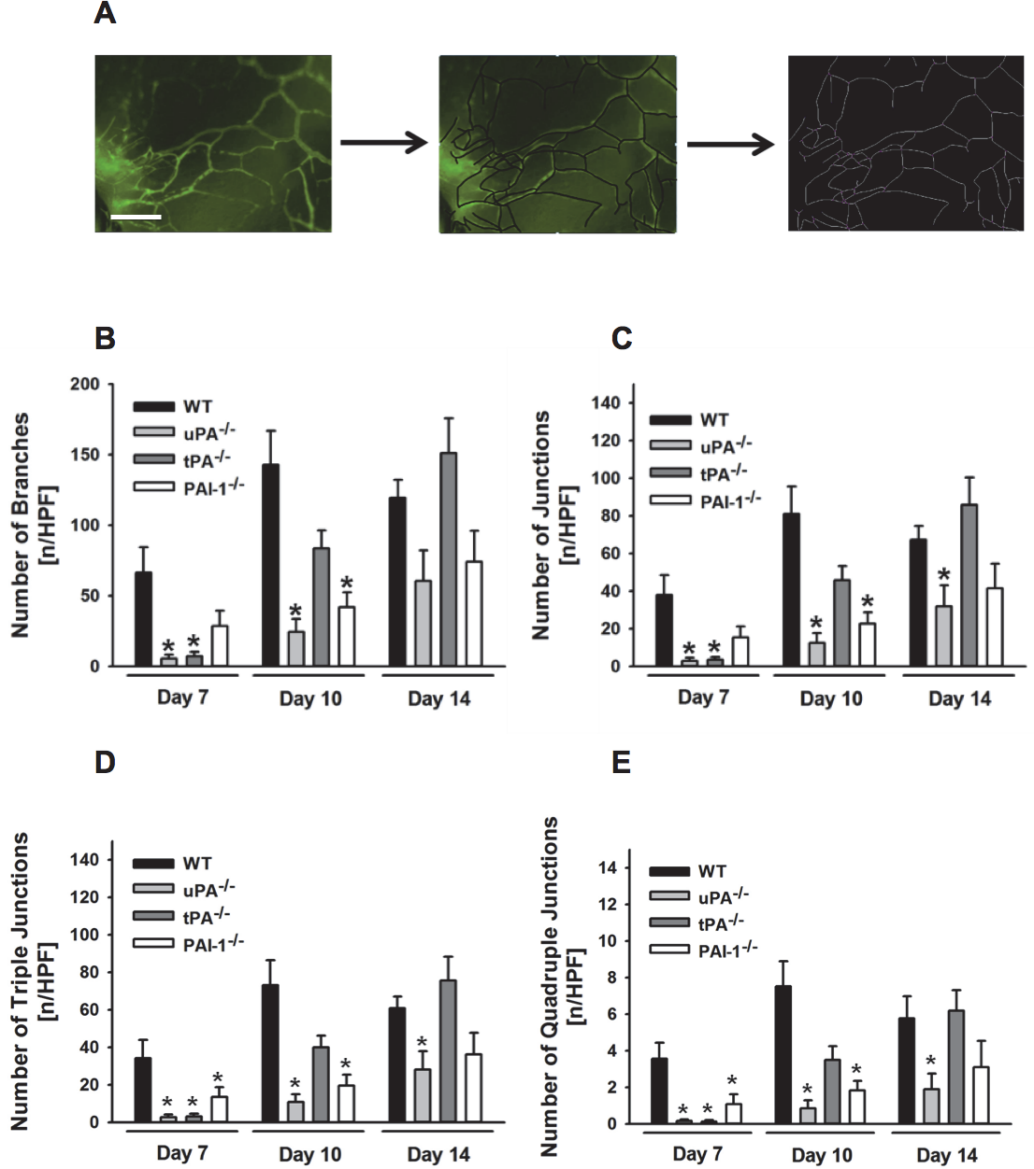
Role of endogenous uPA, tPA, and PAI-1 for vessel network formation in PPE biomaterial. A detailed analysis of the vessel network within the PPE implants in WT as well as in uPA-, tPA-, or PAI-1-deficient mice was performed by software-assisted skeletonizing of the vessel network. Representative images of this analysis in a WT mouse are shown (**A;** scale bar: 100 μm). Panels show results for the number of branches (**B**) and total junctions (**C**) as well as triple (**D**) and quadruple (**E**) junctions in the vessel network (mean ± SEM for n = 6; * p < 0.05 vs. WT).

In order to assess the role of uPA, tPA, and PAI-1 for vascularization of PPE implants in more detail, a comprehensive analysis of the architecture of the newly formed vessel network was performed (Fig. 2A). In the early engraftment phase, a significant reduction in both numbers of vessel branches (Fig. 2B) and vessel junctions (Fig. 2C) was detected in uPA^-/-^ or tPA^-/-^ animals as compared to WT animals. In addition, numbers of triple junctions (Fig. 2D; representing 3 joining vessels) or quadruple junctions (Fig. 2E; representing 4 joining vessels) were significantly reduced in the mutant animals as compared to WT controls. Whereas numbers of vessel branches and junctions were significantly diminished in uPA-deficient animals as compared to WT controls at days 10 and 14 after PPE implantation, these parameters remained unaltered in tPA-deficient animals during this late time period. PAI-1-deficient animals exhibited only a transient significant defect in the formation of vessel branches and junctions at day 10 after implantation, whereas numbers of triple and quadruple vessel network junctions were significantly lower at days 7 and 10 after implantation as compared to WT animals. Noteworthy, the average vessel branch length remained constant over time in all experimental groups (data not shown).

Moreover, the vessel density in the host tissue surrounding the implant material was constant during the entire observation phase and did not differ among experimental groups (**A Table** in S1 File). Furthermore, no significant differences among experimental groups were observed in average diameters, blood flow velocities, and shear rates in microvessels within the implant material and the surrounding tissue at all time points (**B Table** in S1 File).

### Role of uPA, tPA, and PAI-1 for leukocyte-endothelial cell interactions in PPE implants

**Figure 3:**
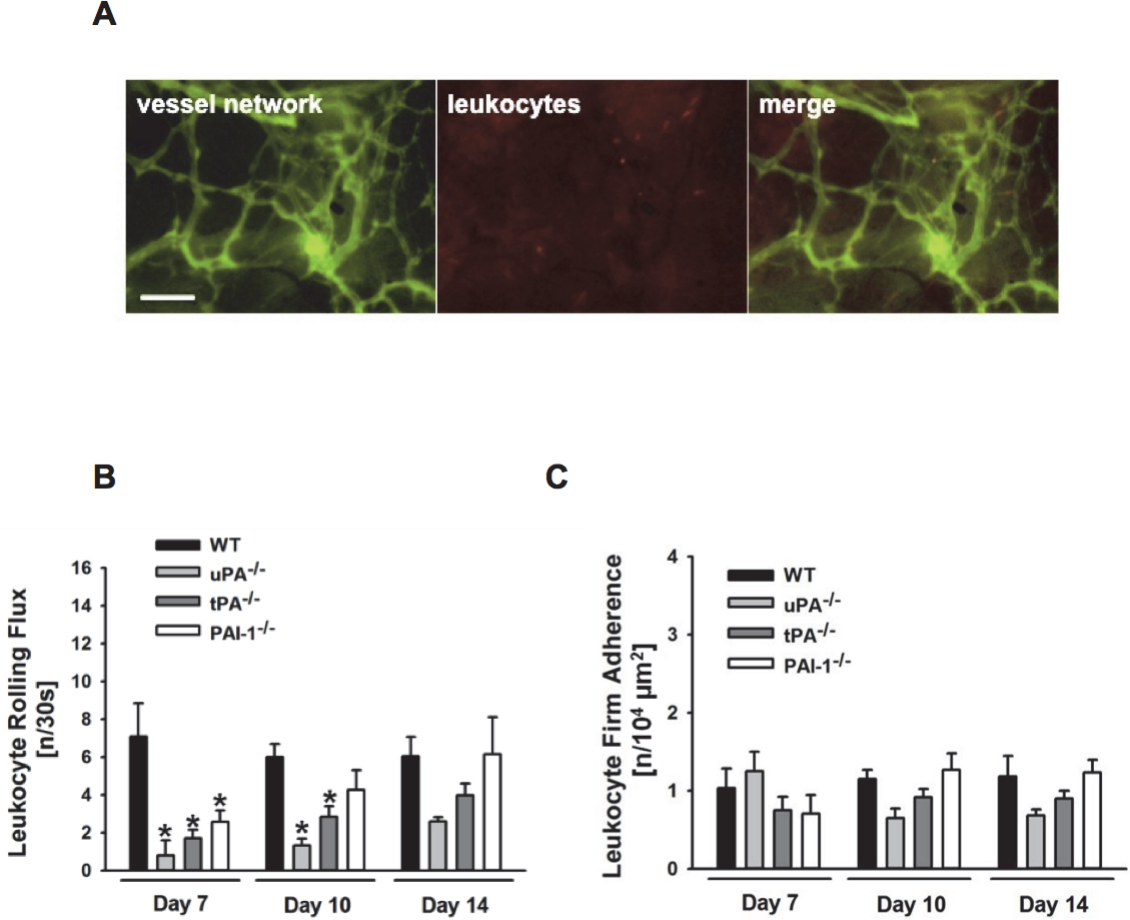
Role of endogenous uPA, tPA, and PAI-1 for leukocyte-endothelial cell interactions in PPE biomaterial. Interactions of fluorescence-labeled leukocytes and endothelial cells in newly formed microvessels within the PPE implants in WT as well as in uPA-, tPA-, or PAI-1-deficient mice were analyzed by *in vivo* fluorescence microscopy. Representative *in vivo* microscopy images of these processes in a WT mouse are shown (**A**; scale bar: 100 μm). Panels show results for the number of intravascularly rolling (**B**) and adherent (**C**) leukocytes (mean ± SEM for n = 6; * p < 0.05 vs. WT).

Leukocyte recruitment is thought to play a critical role in the engraftment of PPE biomaterial [11]. Using *in vivo* fluorescence microscopy, interactions of fluorescence-labeled leukocytes with endothelial cells were analyzed in the newly formed microvasculature within the PPE implants (Fig. 3A). Upon biomaterial implantation, the number of leukocyte-endothelial cell interactions in newly formed microvessels of the implant was low. Whereas in the early engraftment phase the number of intravascularly rolling leukocytes was significantly diminished in microvessels of implants in uPA^-/-^, tPA^-/-^, or PAI-1^-/-^ animals (Fig. 3B), the number of firmly adherent leukocytes remained unchanged (Fig. 3C).

No significant differences in leukocyte-endothelial cell interactions were observed in the microvasculature of the host tissue surrounding the implant material (**A Table** in S1 File).

### Role of uPA, tPA, and PAI-1 for mechanical tissue integration of PPE implants

**Figure 4:**
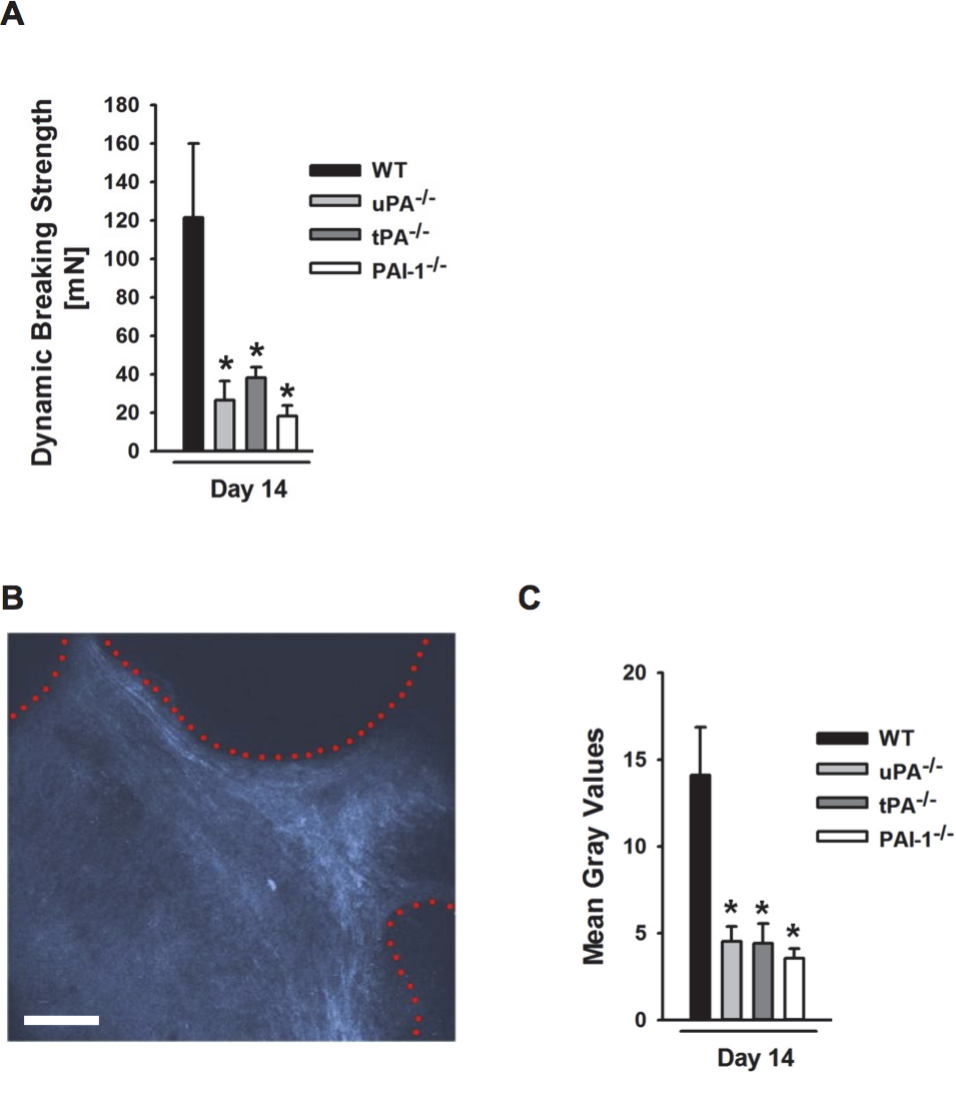
Role of endogenous uPA, tPA, and PAI-1 for mechanical tissue integration of PPE biomaterial. Panel (**A**) shows results for the disintegration force (mean ± SEM for n = 6; * p < 0.05 vs. WT). The disintegration force required for mechanical removal of the implant out of the host tissue was determined in WT as well as in uPA-, tPA-, or PAI-1-deficient mice. Collagen deposition within the PPE biomaterial was analyzed *ex vivo* in implants from WT as well as from uPA-, tPA-, or PAI-1-deficient mice by second harmonic imaging microscopy. A representative image from a WT mouse is shown (**B**; scale bar: 100 μm; red dotted lines delineate pores of the implant material, in which collagen (white) is deposited). Panel (**C**) shows quantitative results for the second harmonic signal (mean ± SEM for n = 4; * p < 0.05 vs. WT).

As a measure of functional tissue integration of PPE implants, the disintegration force required for the mechanical removal of the implant out of the host tissue was assessed at the end of each experiment on day 14 after implantation. In our experiments, the required disintegration force was significantly lower in uPA^-/-^, tPA^-/-^, or PAI-1^-/-^ animals as compared to WT control animals (Fig. 4A).

Mechanical tissue integration of PPE biomaterial is established through the formation of a tight collagen network within the implant that is connected to the surrounding tissue. As a measure of collagen deposition within the implant material, the second harmonic signal (visualizing collagen) was analyzed by using multi-photon microscopy (Fig. 4B). In our experiments, the second harmonic signal in implants from uPA^-/-^, tPA^-/-^, or PAI-1^-/-^ animals was significantly lower than in WT controls (Fig. 4C).

### Chemotactic effect of recombinant uPA, tPA, and PAI-1 on microvascular endothelial cells

**Figure 5:**
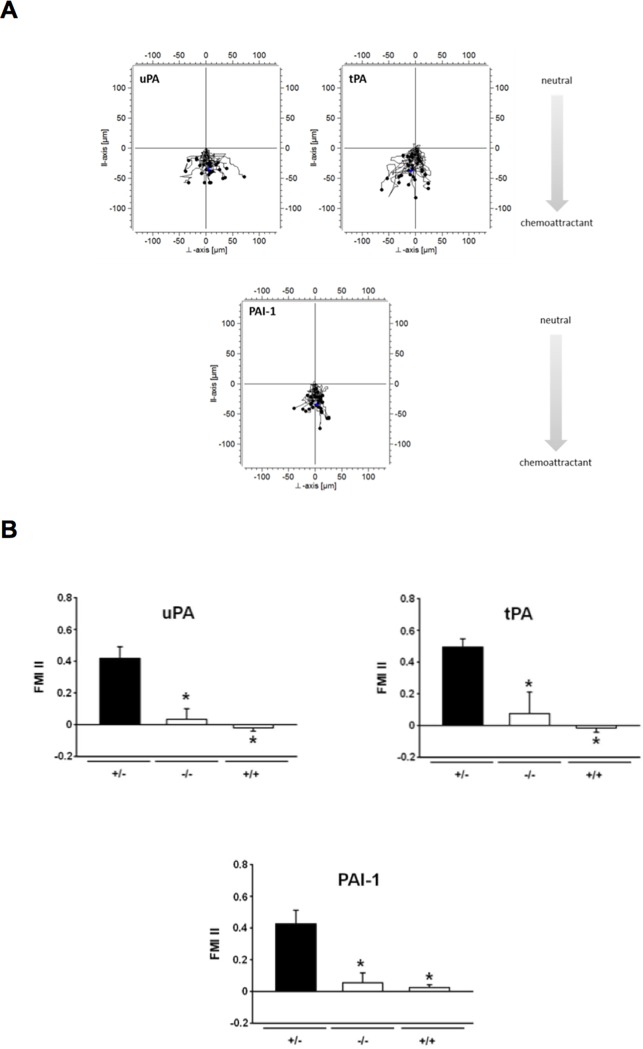
Effect of uPA, tPA, and PAI-1 on migration of endothelial cells. The effect of recombinant uPA, tPA, and PAI-1 on migration of endothelial cells was analyzed *in vitro*. Representative migration plots are shown (**A**). Panels show results for the forward migration index (FMI) of migrating endothelial cells upon exposure to recombinant uPA, tPA, or PAI-1 (**B**; mean ± SEM for n = 3; * p < 0.05 vs. +/-).

Since uPA-, tPA-, and PAI-1-deficiency was associated with reduced vascularization of PPE biomaterial, we hypothesized that these effects are due to the ability of the different components of the plasminogen activation system to recruit endothelial cells. As a measure of the chemotactic effect of uPA, tPA, or PAI-1 on endothelial cells, the forward migration index (FMI) for migrating endothelial cells was determined in an *in vitro* assay (Fig. 5A). Upon exposure to uPA, tPA, or PAI-1, the FMI for migrating endothelial cells was significantly higher than in control experiments (Fig. 5B).

### Effect of surface coating with recombinant uPA, tPA, or PAI-1 on tissue integration of PPE implants

**Figure 6:**
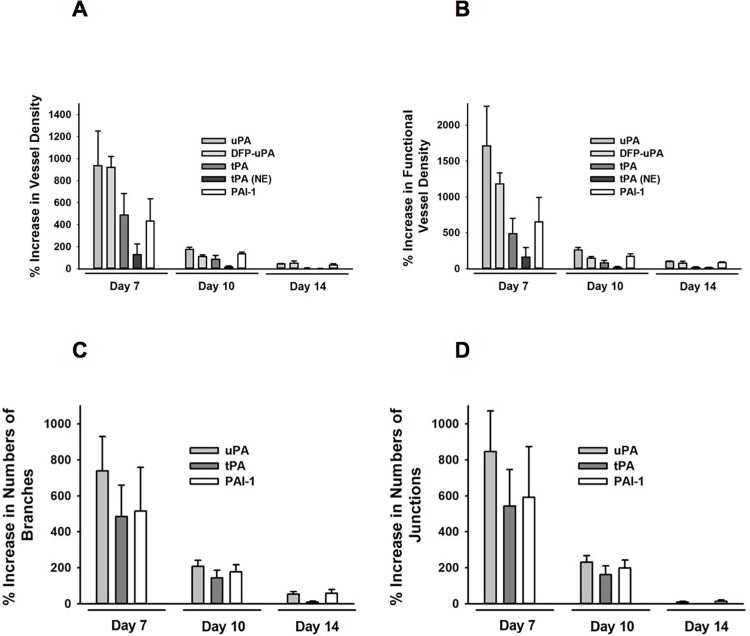
Effect of surface coating of implants with recombinant uPA, tPA, or PAI-1 on vascularization of PPE biomaterial. Vascularization of PPE implants, which were coated with recombinant uPA, tPA, or PAI-1 embedded in Matrigel, was analyzed by *in vivo* fluorescence microscopy in WT mice. Panels show results for the relative increase in absolute (**A**) and functional (**B**) vessel density within the implant as well as for the increase in the number of branches (**C**) and junctions (**D**) in the vessel network as compared to Matrigel-coated control implants (mean ± SEM for n = 5 – 6).

To translate our *in vitro* results in a clinically more relevant situation, PPE biomaterial was coated with recombinant murine uPA, tPA, or PAI-1 embedded in Matrigel and implanted into dorsal skinfold chambers of WT mice. Surface coating with uPA and–to a lesser degree–with tPA or PAI-1 resulted in increased absolute (Fig. 6A) and functional (Fig. 6B) vessel density in PPE implants in the early engraftment phase as compared to Matrigel-coated control implants.

A more detailed analysis revealed that surface coating of PPE implants with recombinant uPA, tPA, or PAI-1 resulted in a significant elevation in numbers of vessel branches (Fig. 6C) and vessel junctions in the vessel network of PPE implants (Fig. 6D) as compared to control implants. No significant differences were observed in vessel diameters, blood flow velocities, and shear rates among experimental groups (**C Table** in S1 File). Furthermore, surface coating with recombinant uPA, tPA, or PAI-1 had no effect on leukocyte-endothelial cell interactions in microvessels within the implant material (**C Table** in S1 File) or the surrounding host tissue (**D Table** in S1 File). Moreover, the vessel density of the surrounding host tissue remained comparable during the entire observation period among experimental groups (**D Table** in S1 File). Similarly, mechanical tissue integration of the implant material remained unaltered upon surface coating with recombinant uPA, tPA, or PAI-1 (**E Table** in S1 File).

### Effect of surface coating with non-catalytically active uPA or tPA proteins on vascularization of PPE implants

**Figure 7:**
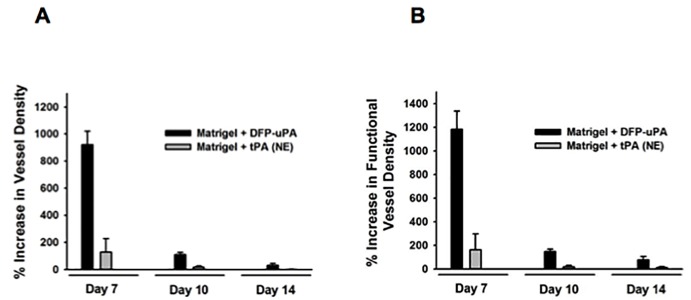
Effect of surface coating of implants with DFP-uPA or NE-tPA on vascularization of PPE biomaterial. Vascularization of PPE implants, which were coated with DFP-uPA or NE-tPA embedded in Matrigel, was analyzed by *in vivo* fluorescence microscopy in WT mice. Panels show results for the relative increase in absolute (**A**) and functional (**B**) vessel density within the implant as compared to Matrigel-coated control implants (mean ± SEM for n = 5 – 6).

To characterize the functional relevance of the catalytic properties of uPA and tPA for early vascularization of PPE biomaterial, implants were coated with DFP-uPA (in which the catalytic activity of uPA is inhibited by diisopropyl fluorophosphate (DFP)) or NE-tPA (which represents a tPA mutant protein completely lacking its catalytic activity) proteins. Surface coating with DFP-uPA resulted in a significant increase in the absolute (Fig. 7A) and functional (Fig. 7B) vessel density in the implant material as compared to control implants. This increase was comparable to implants coated with catalytically active uPA during the early integration phase. In contrast, surface coating with NE-tPA barely enhanced the absolute or functional vessel density in PPE biomaterial. Noteworthy, biomaterial coating with NE-tPA can therefore also be considered as an additional negative control in our experiments.

As confirmed before by zymography, there was no proteolytic activity of DFP-uPA detectable (data not shown; [37]).

## Discussion

Rapid fibrovascularization is a prerequisite for successful engraftment of biomaterials [10, 11]. In addition to their well-known roles in fibrinolysis, the plasminogen activators uPA and tPA as well as their inhibitor PAI-1 are increasingly recognized as individual and autonomous mediators in non-fibrinolytic processes, including cell adhesion, migration, and proliferation [22–30]. Since these events are critical for the fibrovascularization of biomaterial [10, 11], we hypothesized that the components of the plasminogen activation system contribute to the functional biointegration of PPE implants.

In a first approach, immunostaining and confocal microscopy were used to evaluate whether the major components of the plasminogen activation system are present in engrafted PPE implants. Here, we show that uPA, tPA, and PAI-1 are expressed by microvascular endothelial cells and deposited in and around the newly formed microvessels within the implant. These observations complement previous reports documenting that uPA, tPA, and PAI-1 are synthesized by cultured endothelial cells [19–21].

The rapid establishment of a reliable vessel network within implanted biomaterial is strictly required for the survival of oxygen dependent structures as well as for the prevention of implant infection and rejection [38,39]. To analyze the functional relevance of the plasminogen activation system for the vascularization of PPE biomaterial, *in vivo* epi-fluorescence microscopy was employed in implants engrafted in dorsal skinfold chambers of WT and appropriate mutant mice. Our data indicate that the major components of the plasminogen activation system selectively, but differentially support the vascularization process of this biomaterial without effects on the existing microvasculature of the surrounding host tissue: Whereas tPA is predominantly involved in the initial stages of microvessel network formation within PPE implants, uPA additionally exhibits sustained effects on vascularization of this biomaterial. Noteworthy, PAI-1 which serves as the principal inhibitor of the plasminogen activators in fibrinolysis, promotes the formation of the vessel network within the implant differentially at various stages of PPE implant integration. Our results extend previous observations implicating uPA [23], tPA [40], and PAI-1 [41] in vasculo-/angiogenesis.

Taking a closer look on the architecture of the developing vessel network within the implant, we found that uPA, tPA, and PAI-1 predominantly facilitate branch formation, but not length extension of single vessel segments in the growing network suggesting that these endogenously released proteins are particularly involved in the maturation of the vessel network within the biomaterial. This is an interesting finding which might be related to non-fibrinolytic properties of these proteins that have been identified to promote proliferation of endothelial cells [42–44].

In addition to its non-fibrinolytic effects on endothelial cells, uPA, tPA, and PAI-1 have been reported to contribute to the regulation of leukocyte recruitment under severe inflammatory conditions including ischemia-reperfusion injury [37,45–47] or sepsis [48]. Upon biomaterial implantation, however, only a ‘moderate’ inflammatory response arises which might explain that deficiency of the major components of the plasminogen activation system did not substantially alter leukocyte-endothelial cell interactions within the implant or in the surrounding host tissue in our experiments.

Along with the vascularization of PPE implants, fibroblasts invade the pores of this biomaterial and secrete collagen. This leads to the formation of a dense collagen network that ultimately ties the implant to the surrounding host tissue. Using second harmonic generation multi-photon microscopy, we were able to demonstrate that the major components of the plasminogen activation system are critically involved in the development of this collagen network. In this context, uPA, tPA, and PAI-1 are thought to induce proliferation of fibroblasts and stimulate collagen production [28–30]. Accordingly, the force needed for mechanical removal of the implant out of the host tissue was significantly reduced in uPA-, tPA-, or PAI-1-deficient animals. Thus, our experimental data reveal that the major components of the plasminogen activation system play a crucial role for the tissue integration of PPE implants through common and distinct effects on fibrovascularization of this biomaterial.

Despite its excellent biocompatibility, there are applications which are less favorable for PPE implant integration, such as rhinoplasty and reconstructive nose surgery [8] as well as reconstructions in irradiated or scar tissue [9], where blood supply is weak. Previous attempts to improve tissue integration of biomaterials included coating of implants with autologous material, such as chondrocytes [10,49], adipocytes [50], adipose tissue derived microvascular fragments [51], or acellular dermis [52] as well as with specific growth factors, e. g. basic fibroblast growth factor [bFGF; [53–55]] or vascular endothelial growth factor [VEGF; [34]]. Less specific, but already in clinical use, PPE coated with oxidized cellulose polymers and the patient’s blood (which contains uPA, tPA, and PAI-1) is successfully employed in reconstructive nose surgery [56,57]. Together with our actual findings on the role of the plasminogen activation system for the engraftment of PPE implants, we consequently hypothesized that surface coating with recombinant uPA, tPA, or PAI-1 supports the tissue integration of this biomaterial. In a separate set of *in vitro* experiments, we found that uPA, tPA, and PAI-1 potently attract endothelial cells. These results are in line with recent observations involving the plasminogen activation system in VEGF (vascular endothelial growth factor)-dependent vessel growth which represents one of the most prominent pathways in angiogenesis [27]. To translate these results into a clinically more relevant context, PPE implants were embedded in Matrigel supplemented with components of the plasminogen activation system and engrafted in dorsal skinfold chambers of WT mice (Fig. 6). Surface coating with recombinant uPA, tPA, or PAI-1 lead to an accelerated vascularization of PPE implants predominantly in the initial engraftment phase. Whereas tPA particularly improved initial implant vascularization (as compared to uPA or PAI-1), surface coating with recombinant PAI-1 still showed beneficial effects on vessel network formation within the implant at late stages of the engraftment process. Notably, surface coating with recombinant uPA exhibited the most striking effect on initial implant vascularization (as compared to tPA or PAI-1) that persisted (at lower levels) over the entire experimental period.

In recent studies, the plasminogen activators have been supposed to mediate their non-fibrinolytic properties via catalytic and non-catalytic effects [24–27,36]. Towards a more mechanistic understanding of these uPA- and tPA-dependent engraftment processes, we coated PPE implants with modified tPA or uPA proteins lacking their catalytic activity. Our experimental data indicate that tPA promotes the vascularization of PPE implants mainly via its proteolytic properties, whereas uPA predominantly employs its non-proteolytic properties to support vessel network formation in this biomaterial. Since plasmin has previously been implicated in tissue integration of biomaterial implants [36], our data suggest that the reported effect of plasmin on biomaterial engraftment is predominantly mediated via tPA-dependent proteolytical activation of plasminogen. In this context it is interesting that tPA-dependent activation of plasminogen has recently been found to contribute to biological processes different from vessel network formation including leukocyte recruitment to inflamed tissue [47].

In contrast to our findings on vascularization of PPE implants, however, surface coating with components of the plasminogen activation system did not further enhance collagen deposition and subsequent mechanical tissue integration of this biomaterial in our experiments. These observations might be explained by the already outstanding engraftment properties of PPE in densely vascularized and healthy host tissue. Under unfavorable implant conditions, however, surface coating with components of the plasminogen activation system might provide a novel, tailor-made strategy for the acceleration of biomaterial vascularization at different stages of the engraftment process which might prevent implant infection and rejection.

## Conclusions

Collectively, our experimental data demonstrate that endogenously released uPA, tPA, and PAI-1 contribute to the functional biointegration of PPE implants via common and distinct effects on implant fibrovascularization: Whereas tPA predominantly facilitates the initial vessel network formation in PPE biomaterial, uPA additionally exhibits sustained effects on vascularization of these implants. Notably, PAI-1, which serves as the principal inhibitor of tPA and uPA in fibrinolysis, supports vascularization of PPE biomaterial differentially at various stages of the engraftment process. Furthermore, the major components of the plasminogen activation system were found to be crucially involved in collagen deposition within PPE biomaterial thereby promoting mechanical tissue integration of the implants. Interestingly enough, uPA, tPA, and PAI-1 exhibited strong chemotactic effects on endothelial cells. Accordingly, surface coating with recombinant uPA (mainly via its non-proteolytic properties) and–to a lesser degree–with tPA (mainly via its proteolytic properties) or PAI-1 accelerated vascularization of PPE implants. Our experimental findings provide novel insights into the mechanisms underlying tissue integration of biomaterials and propose surface coating with recombinant uPA, tPA, or PAI-1 as a novel promising strategy for accelerating the vascularization of PPE implants under unfavorable conditions.

## Supporting Information

S1 File Contains the following files: A Table. Microhemodynamic parameters and leukocyte responses in host tissue. Microhemodynamic parameters and leukocyte responses in the host tissue were determined as detailed in *Material and Methods* (mean ± SEM for n = 5 – 6). **B Table. Microhemodynamic parameters in implants.** Microhemodynamic parameters in implants were obtained as detailed in *Material and Methods* (mean ± SEM for n = 5 – 6). **C Table. Microhemodynamic parameters and leukocyte responses in coated implants.** Microhemodynamic parameters and leukocyte responses in coated implants were determined as detailed in *Material and Methods* (mean ± SEM for n = 5–6). **D Table. Vessel density and leukocyte responses in host tissue upon engraftment of coated implants.** Vessel density and leukocyte responses in the host tissue were determined upon engraftment of coated implants as detailed in *Material and Methods* (mean ± SEM for n = 5–6). **E Table. Mechanical tissue integration of coated PPE biomaterial.** The disintegration force required for mechanical removal of implants coated with recombinant murine uPA, tPA, PAI-1, DFP-uPA, or NE-tPA out of the host tissue was determined upon engraftment of coated implants as detailed in *Material and Methods* (mean ± SEM for n = 5–6).
(DOCX)
