# Supplementary material for: Components of the Plasminogen Activation System Promote Engraftment of Porous Polyethylene Biomaterial via Common and Distinct Effects
Source: PLoS One. 2015 Feb 6;10(2):e0116883. doi: 10.1371/journal.pone.0116883 (PMC4319722; doi:10.1371/journal.pone.0116883)
Supplement: S1 File — Microhemodynamic parameters and leukocyte responses in the host tissue were determined as detailed in Material and Methods (mean ± SEM for n = 5 – 6). B Table. Microhemodynamic parameters in implants. Microhemodynamic parameters in implants were obtained as detailed in Material and Methods (mean ± SEM for n = 5 – 6). C Table. Microhemodynamic parameters and leukocyte responses in coated implants. Microhemodynamic parameters and leukocyte responses in coated implants were determined as detailed in Material and Methods (mean ± SEM for n = 5–6). D Table. Vessel density and leukocyte responses in host tissue upon engraftment of coated implants. Vessel density and leukocyte responses in the host tissue were determined upon engraftment of coated implants as detailed in Material and Methods (mean ± SEM for n = 5–6). E Table. Mechanical tissue integration of coated PPE biomaterial. The disintegration force required for mechanical removal of implants coated with recombinant murine uPA, tPA, PAI-1, DFP-uPA, or NE-tPA out of the host tissue was determined upon engraftment of coated implants as detailed in Material and Methods (mean ± SEM for n = 5–6). (DOCX) [file pone.0116883.s001.docx]

**Supplemental Tables**

**A Table Microhemodynamic parameters and leukocyte responses in host tissue.** Microhemodynamic parameters and leukocyte responses in the host tissue were determined as detailed in *Material and Methods* (mean ± SEM for n = 5 - 6).

**B Table. Microhemodynamic parameters in implants.** Microhemodynamic parameters in implants were obtained as detailed in *Material and Methods* (mean ± SEM for n = 5 - 6).

**C Table. Microhemodynamic parameters and leukocyte responses in coated implants.** Microhemodynamic parameters and leukocyte responses in coated implants were determined as detailed in *Material and Methods* (mean ± SEM for n = 5 - 6).

**D Table. Vessel density and leukocyte responses in host tissue upon engraftment of coated implants.** Vessel density and leukocyte responses in the host tissue were determined upon engraftment of coated implants as detailed in *Material and Methods* (mean ± SEM for n = 5 - 6).

**E Table. Mechanical tissue integration of coated PPE biomaterial.** The disintegration force required for mechanical removal of implants coated with recombinant murine uPA, tPA, PAI-1, DFP-uPA, or NE-tPA out of the host tissue was determined upon engraftment of coated implants as detailed in *Material and Methods* (mean ± SEM for n = 5 - 6).

**Supplemental Table A**

|  | | **Day 7** | **Day 10** | **Day 14** |
| --- | --- | --- | --- | --- |
|  | |  |  |  |
| **Vessel Density Host Tissue**  **[cm/cm²]** | **WT** | 247.3 ± 19.5 | 265.9 ± 40.3 | 283.1 ± 17.7 |
|  | **uPA^-/-^** | 250.6 ± 15.3 | 255.2 ± 16.3 | 253.9 ± 19.4 |
|  | **tPA^-/-^** | 261.3 ± 13.2 | 282.7 ± 19.3 | 305.5 ± 28.5 |
|  | **PAI-1^-/-^** | 251.3 ± 18.0 | 271.5 ± 13.3 | 300.2 ± 18.2 |
|  |  |  |  |  |
| **Vessel Diameter Host Tissue**  **[µm]** | **WT** | 20.3 ± 0.5 | 20.5 ± 1.2 | 19.6 ± 1.0 |
|  | **uPA^-/-^** | 20.4 ± 0.9 | 19.2 ± 2.0 | 18.8 ± 1.6 |
|  | **tPA^-/-^** | 17.9 ± 1.3 | 18.1 ± 0.9 | 17.2 ± 1.0 |
|  | **PAI-1^-/-^** | 21.6 ± 1.1 | 18.4 ± 2.3 | 17.8 ± 2.2 |
|  |  |  |  |  |
| **Blood Flow Velocity**  **Host Tissue**  **[mm/s]** | **WT** | 0.183 ± 0.011 | 0.186 ± 0.028 | 0.184 ± 0.023 |
|  | **uPA^-/-^** | 0.221 ± 0.089 | 0.160 ± 0.024 | 0.163 ± 0.039 |
|  | **tPA^-/-^** | 0.154 ± 0.024 | 0.182 ± 0.033 | 0.179 ± 0.053 |
|  | **PAI-1^-/-^** | 0.184 ± 0.016 | 0.196 ± 0.022 | 0.128 ± 0.021 |

| **Shear rate**  **[s^-1^]** | **WT** | 354.8 ± 21.0 | 357.4 ± 53.7 | 367.6 ± 36.6 |
| --- | --- | --- | --- | --- |
|  | **uPA^-/-^** | 435.8 ± 110.1 | 342.4 ± 55.2 | 334.9 ± 36.8 |
|  | **tPA^-/-^** | 338.8 ± 37.4 | 392.5 ± 48.1 | 409.9 ± 103.6 |
|  | **PAI-1^-/-^** | 335.6 ± 26.2 | 436.9 ± 51.7 | 296.4 ± 56.5 |
|  |  |  |  |  |
| **Leukocyte Rolling Host Tissue**  **[n/30s]** | **WT** | 7.4 ± 1.5 | 5.3 ± 1.2 | 4.6 ± 0.7 |
|  | **uPA^-/-^** | 7.3 ± 1.8 | 5.1 ± 0.8 | 4.2 ± 0.5 |
|  | **tPA^-/-^** | 4.6 ± 1.3 | 3.5 ± 0.3 | 3.6 ± 0.2 |
|  | **PAI-1^-/-^** | 5.8 ± 1.6 | 3.8 ± 0.6 | 4.9 ± 1.2 |
|  |  |  |  |  |
| **Leukocyte Adherence Host Tissue [n/10^4^ µm^2^]** | **WT** | 1.5 ± 0.2 | 1.2 ± 0.4 | 1.2 ± 0.1 |
|  | **uPA^-/-^** | 1.6 ± 0.4 | 1.1 ± 0.2 | 0.9 ± 0.2 |
|  | **tPA^-/-^** | 1.5 ± 0.4 | 1.1 ± 0.2 | 0.9 ± 0.1 |
|  | **PAI-1^-/-^** | 1.2 ± 0.1 | 1.1 ± 0.1 | 0.9 ± 0.2 |

**Supplemental Table B**

|  | | **Day 7** | **Day 10** | **Day 14** |
| --- | --- | --- | --- | --- |
|  |  |  |  |  |
| **Vessel Diameter Implant**  **[µm]** | **WT** | 17.0 ± 1.1 | 17.6 ± 0.7 | 16.3 ± 0.5 |
|  | **uPA^-/-^** | 16.5 ± 0.8 | 16.6 ± 1.4 | 15.6 ± 1.8 |
|  | **tPA^-/-^** | 15.1 ± 1.0 | 16.2 ± 0.7 | 14.8 ± 0.7 |
|  | **PAI-1^-/-^** | 15.2 ± 1.3 | 15.8 ± 1.0 | 16.7 ± 1.3 |
|  |  |  |  |  |
| **Blood Flow Velocity Implant**  **[mm/s]** | **WT** | 0.117 ± 0.018 | 0.155 ± 0.022 | 0.148 ± 0.009 |
|  | **uPA^-/-^** | 0.143 ± 0.011 | 0.114 ± 0.041 | 0.124 ± 0.017 |
|  | **tPA^-/-^** | 0.110 ± 0.030 | 0.132 ± 0.012 | 0.143 ± 0.034 |
|  | **PAI-1^-/-^** | 0.123 ± 0.016 | 0.178 ± 0.032 | 0.131 ± 0.015 |

| **shear rate**  **[s^-1^]** | | **WT** | 268.3 ± 39.0 | | 350.0 ± 53.5 | 355.3 ± 23.8 |
| --- | --- | --- | --- | --- | --- | --- |
|  |  | **uPA^-/-^** | 254.8 ± 71.1 | | 272.8 ± 54.6 | 312.9 ± 21.4 |
|  |  | **tPA^-/-^** | 287.8 ± 70.5 | | 318.2 ± 14.5 | 385.0 ± 41.4 |
|  |  | **PAI-1^-/-^** | 251.4 ± 64.6 | | 454.4 ± 95.5 | 317.5 ± 45.6 |
|  |  | | |  |  |  |

**Supplemental Table C**

|  | | **Day 7** | **Day 10** | **Day 14** |
| --- | --- | --- | --- | --- |
|  | |  |  |  |
| **Vessel Diameter Implant**  **[µm]** | **MG** | 15.1 ± 0.7 | 17.1 ± 1.3 | 16.2 ± 0.3 |
|  | **MG+uPA** | 16.5 ± 0.8 | 16.9 ± 0.8 | 16.0 ± 0.7 |
|  | **MG+tPA** | 15.1 ± 0.7 | 16.1 ± 0.7 | 16.6 ± 0.6 |
|  | **MG+PAI-1** | 15.9 ± 0.5 | 18.0 ± 0.6 | 16.0 ± 0.9 |
|  |  |  |  |  |
| **Blood Flow Velocity Implant**  **[mm/s]** | **MG** | 0.095 ± 0.004 | 0.158 ± 0.016 | 0.163 ± 0.037 |
|  | **MG+uPA** | 0.140 ± 0.032 | 0.171 ± 0.019 | 0.150 ± 0.019 |
|  | **MG+tPA** | 0.117 ± 0.022 | 0.166 ± 0.017 | 0.151 ± 0.020 |
|  | **MG+PAI-1** | 0.129 ± 0.028 | 0.153 ± 0.014 | 0.140 ± 0.016 |
|  |  |  |  |  |
| **shear rate**  **[s^-1^]** | **MG** | 249.6 ± 20.2 | 368.7 ± 43.1 | 386.7 ± 97.1 |
|  | **MG+uPA** | 337.9 ± 86.7 | 399.7 ± 45.9 | 368.7 ± 38.2 |
|  | **MG+tPA** | 300.3 ± 54.1 | 407.2 ± 43.2 | 356.8 ± 42.4 |
|  | **MG+PAI-1** | 312.2 ± 56.9 | 334.4 ± 28.9 | 341.9 ± 30.9 |
|  |  |  |  |  |
| **Leukocyte Rolling Implant**  **[n/30s]** | **MG** | 2.2 ± 0.6 | 2.0 ± 0.6 | 3.3 ± 0.3 |
|  | **MG+uPA** | 1.9 ± 0.4 | 2.7 ± 0.4 | 3.8 ± 0.4 |
|  | **MG+tPA** | 1.9 ± 0.5 | 1.8 ± 0.4 | 2.6 ± 0.4 |
|  | **MG+PAI-1** | 1.8 ± 0.3 | 2.7 ± 0.5 | 2.6 ± 0.5 |
| **Leukocyte** | **MG** | 0.7 ± 0.3 | 0.8 ± 0.2 | 1.1 ± 0.1 |
| **Adherence** | **MG+uPA** | 1.0 ± 0.2 | 0.9 ± 0.2 | 1.2 ± 0.1 |
| **Implant** | **MG+tPA** | 0.9 ± 0.2 | 0.9 ± 0.1 | 0.9 ± 0.1 |
| **[n/10^4^ µm^2^]** | **MG+PAI-1** | 0.9 ± 0.3 | 0.9 ± 0.1 | 0.8 ± 0.1 |

**Supplemental Table D**

|  | | **Day 7** | **Day 10** | **Day 14** |
| --- | --- | --- | --- | --- |
|  |  |  |  |  |
| **Vessel Density Host Tissue**  **[cm/cm²]** | **MG** | 309.2 ± 18.3 | 305.1 ± 15.7 | 328.9 ± 17.4 |
|  | **MG+uPA** | 294.1 ± 16.1 | 311.2 ± 14.2 | 311.2 ± 9.1 |
|  | **MG+tPA** | 278.0 ± 16.1 | 302.6 ± 17.7 | 296.3 ± 10.0 |
|  | **MG+PAI-1** | 288.3 ± 10.0 | 291.9 ± 15.2 | 319.1 ± 17.8 |
|  |  |  |  |  |
| **Leukocyte Rolling Host Tissue**  **[n/30s]** | **MG** | 3.1 ± 0.6 | 3.1 ± 0.7 | 4.4 ± 0.9 |
|  | **MG+uPA** | 3.8 ± 0.5 | 4.2 ± 1.1 | 5.3 ± 1.1 |
|  | **MG+tPA** | 2.4 ± 0.5 | 2.3 ± 0.2 | 3.1 ± 0.2 |
|  | **MG+PAI-1** | 3.6 ± 0.6 | 3.5 ± 0.9 | 3.1 ± 0.5 |
|  |  |  |  |  |
| **Leukocyte Adherence Host Tissue [n/10^4^ µm^2^]** | **MG** | 1.1 ± 0.2 | 1.0 ± 0.2 | 1.0 ± 0.2 |
|  | **MG+uPA** | 1.1 ± 0.2 | 1.3 ± 0.2 | 1.1 ± 0.2 |
|  | **MG+tPA** | 1.1 ± 0.1 | 1.0 ± 0.1 | 1.0 ± 0.1 |
|  | **MG+PAI-1** | 1.4 ± 0.2 | 0.9 ± 0.1 | 1.2 ± 0.1 |

**Supplemental Table E**

|  | | **Day 14** |  |  |
| --- | --- | --- | --- | --- |
|  | |  |  |  |
| **Dynamic Breaking Strength**  **[mN]** | **MG** | 31.7 ±7-0 |  |  |
|  | **MG+uPA** | 50.0 ± 6.3 |  |  |
|  | **MG+tPA** | 41.7 ± 10.1 |  |  |
|  | **MG+PAI-1**  **MG+DFP-uPA**  **MG+NE-tPA** | 50.0 ± 5.8  34.0 ± 6.8  24.0 ± 4.7 |  |  |
